# An Integrative Approach to Selected Species of *Tanacetum* L. (Asteraceae): Insights into Morphology and Phytochemistry

**DOI:** 10.3390/plants13020155

**Published:** 2024-01-05

**Authors:** Claudia Giuliani, Martina Bottoni, Fabrizia Milani, Alberto Spada, Sara Falsini, Alessio Papini, Laura Santagostini, Gelsomina Fico

**Affiliations:** 1 Department of Pharmaceutical Sciences, University of Milan, Via Mangiagalli 25, 20133 Milan, Italy; martina.bottoni@unimi.it (M.B.); fabrizia.milani@unimi.it (F.M.); gelsomina.fico@unimi.it (G.F.); 2 Ghirardi Botanic Garden, Department of Pharmaceutical Sciences, University of Milan, Via Religione 25, Toscolano Maderno, 25088 Brescia, Italy; 3 Department of Agricultural and Environmental Sciences—Production, Landscape, Agroenergy, University of Milan, Via Celoria 2, 20133 Milan, Italy; alberto.spada@unimi.it; 4 Department of Biology, University of Florence, Via Giorgio La Pira, 50121 Florence, Italy; sara.falsini@unifi.it (S.F.); alessio.papini@unifi.it (A.P.); 5 Department of Chemistry, University of Milan, Via Golgi 19, 20133 Milan, Italy; laura.santagostini@unimi.it

**Keywords:** *Tanacetum vulgare*, *Tanacetum parthenium*, *Tanacetum corymbosum*, botanic gardens, glandular trichomes, essential oils, microscopy, GC-MS, Open Science

## Abstract

In this work, we studied *Tanacetum vulgare*, *Tanacetum parthenium*, and *Tanacetum corymbosum* (Asteraceae) cultivated at the Ghirardi Botanic Garden (Toscolano Maderno, Brescia, Northern Italy) of the University of Milan. An integrative research approach was adopted: microscopic and histochemical, with special focus on the secretory structures responsible for the productivity of secondary metabolites; phytochemical, with the analysis of the essential oil (EO) profiles from the air-dried, flowered aerial parts collected in June 2021; bio-ecological, with emphasis, based on literature data, on the ecology and biological activity of the main EO components. In all three species, two basic trichome morphotypes (flagellar non-glandular and biseriate glandular) occurred with different distribution patterns. The glandular ones produced terpenes, along with flavonoids. A high level of chemical variability in the EO compositions emerged, specifically for qualitative data. *T. vulgare* profile was more complex and heterogeneous than those obtained from *T. parthenium* and *T. corymbosum*, with camphor as the predominant compound, followed by farnesol and α-santalone, respectively. Finally, the obtained scientific findings were made available to the visitors of the botanic garden through new dissemination labeling that highlights the “invisible”, microscopic features of the plants, from an Open Science perspective (“Botanic Garden, factories of molecules…work in progress”—Lombardy Region Project Lr. 25/2016, year 2021).

## 1. Introduction

*Tanacetum* L. (Asteraceae) is included within the tribe Anthemideae and comprises about 160 species, many of which have been and still are used as medicinal plants, particularly in folk medicine [1,2,3]. It is a morphologically well-delimited genus; however, its taxonomic treatment appears problematic. Various authors do not agree with a comprehensive picture regarding the species conspectus of the genus [3]. This inconsistent situation poses some problems, especially in the commercial field, since a number of *Tanacetum* species have become important and useful, particularly for essential oil (EO) production [2]. Whereas several studies on the chemosystematics of the Asteraceae in general were undertaken by some researchers ([4,5,6,7] and literature therein), literature shows few attempts to detail the chemical classification of the genus *Tanacetum* [8,9,10,11]. The occurrence of EOs, bitter substances, sesquiterpene lactones, flavonoids, and other constituents [9,12,13,14,15,16,17,18], along with the wide range of the related biological activities, were the subject of previous papers and reviews on *Tanacetum*. Antioxidant [19,20,21], immunomodulatory [22], cytotoxic [20,21], anti-inflammatory, and growth-regulating properties [20,23] were documented, in addition to antifeedant, insecticide, and antimicrobial activities [21,24,25,26].

**Figure 1:**
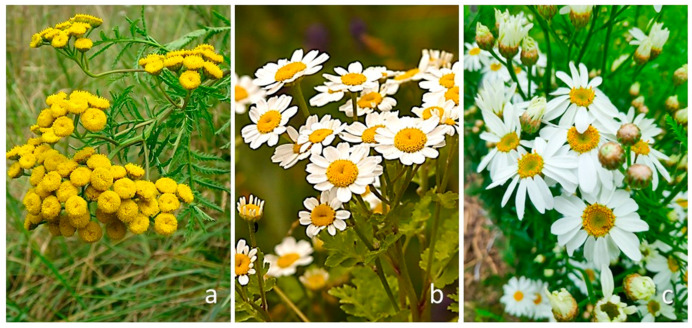
Macrographs showing the examined *Tanacetum* species: (**a**) *Tanacetum vulgare* L., (**b**) *Tanacetum parthenium* (L.) Sch.Bip., (**c**) *Tanacetum corymbosum* (L.) Sch.Bip.

Recently, special attention has been paid to, among others, three species of this genus (Figure 1)—tansy (*Tanacetum vulgare* L.), feverfew (*Tanacetum parthenium* (L.) Sch.Bip.), and corymbflower tansy (*Tanacetum corymbosum* (L.) Sch.Bip.). 

*Tanacetum vulgare* is a perennial herbaceous plant, 60–120 cm high, strongly aromatic, with a creeping and branched, woody rhizome, provided with erect, leafy, striated stems, branched in the upper part. The native range of this species is temperate Eurasia. The leaves are petiolate, alternate, glabrous, with 15–23 pinnate-partite segments with a serrated margin; the lower side of the sheathing cauline leaves show small glands. The compound inflorescences are dense and flattened terminal corymbs with flower heads (diameter = 1 cm) consisting of all tubular yellow-gold-colored florets, having a long pedunculate discoid shape. The peripheral florets, around the margin of the inflorescences, are female with a tridentate corolla, while the central ones are hermaphroditic with a five-toothed corolla. The involucral bracts are lanceolate-obtuse in shape and scarious at the margin, very appressed. The fruits are about 2 mm long achenes with four to five longitudinal ribs, provided with glands; pappus with a small, irregularly furrowed crown [27].

*Tanacetum parthenium* is a glabrescent biennial or perennial herbaceous plant with the peculiar smell of pyrethrum, 30–80 cm high and with a taproot. The native range is South-East Europe to Central Asia and West Himalaya. Stems are erect with reddish streaks, pubescent, with shoots branching out at the distal portion forming a loose corymb. The leaves are alternate, bipinnate: the basal ones early deciduous, the lower cauline ones petiolate, with a lamina (3–4 × 6–9 cm) divided into 5–11 deeply pinnate-partite segments and with marginal and obtuse teeth. Florets are placed on showy flower heads (diameter = 1 cm), equipped with a 2–4 cm peduncle, and with an envelope of angular involucral bracts with a scarious margin. Ray florets are white with linear ligules of 5–10 mm with longitudinal veins and with a bi-trifid truncated apex. Disc florets are tubular and yellow. Fruits are achenes with five to six evident ribs and surmounted by a 0.2–0.3 mm toothed crown [27]. 

*Tanacetum corymbosum* is a perennial, herbaceous plant, 50–120 cm high, with erect, leafy, striated stems. Its native range is Europe to West Asia. Leaves are pinnate and glabrescent; the basal ones are petiolate, oblong–elliptical in shape with pinnate-partite lanceolate segments divided up to the middle of the semi-lamina; the cauline ones are similar but sessile; the upper side is greyish green, almost velvety. The 3–5.5 cm large flower heads are gathered in irregular terminal corymbs, carried by long peduncles; involucral bracts are dark brown in color, arranged in three series, lanceolate with a narrow-rounded apex and scarious brownish margin; ray florets (3–4.5 × 12–20 mm) with white ligules; disc florets yellow. The fruits are achenes with 5five to six ribs and a short crown [27].

They belong to different sections [2,28] of *Tanacetum* L.: the first is included within *Tanacetum* sect. *Tanacetum* and the others within *Tanacetum* sect. *Pyrethrum*, mainly based on the inflorescence features such as white- and red-rayed inflorescence species (former genus *Pyrethrum*), as opposed to the discoid, disciform, or yellow-rayed ones (*Tanacetum* L. *s. s.*). The previous treatment of *Pyrethrum* Zinn. as an independent genus was particularly important for the content in pyrethrins of several species, among the most important insecticidal compounds dating back to Persia, about 400 BC. The strong and aromatic scent of the species considered here is mainly due to high concentrations of volatile terpenes, constituents of their EOs and produced by glandular trichomes, especially on leaves and inflorescences [29]. 

Concerning literature data on these secretory structures, previous investigations were focused on the morphotypes, and the localization of trichomes on the vegetative and reproductive organs of *T. vulgare* and *T. parthenium*, by means of both light and scanning electronic microscopy [30,31,32,33]. Histochemical reports were restricted to the leaf *indumentum* of *T. vulgare* grown in vitro [33]. Contributions on *T. corymbosum* are lacking.

Taking into consideration previous phytochemical works on EOs and biological activities of these species, a richer literature exists for tansy and feverfew, whereas limited information is available for corymbflower tansy. 

Many studies on tansy from diverse geographical regions showed that this species displays a noteworthy infraspecific variability in the terpene constituents of the leaf and inflorescence EOs, revealing the existence of different chemotypes in which the concentration of the main component varied between 40.0% and 70.0% [13,14,34,35,36,37]. Tansy EO exhibits antioxidant, antihypertensive, diuretic, and anthelmintic effects, as well as antibacterial, antifungal, acaricidal, and repellent activities [13,14,34,35,36,37]. 

The chemistry of feverfew EOs was well defined: the profiles were highly variable in terms of total number of compounds, dominant components, and their relative percentages [13,14,29,30,31,32,33,34,35,36,37,38,39,40,41,42,43]. It is listed in the European Pharmacopeia as a traditional herbal remedy used for prophylaxis of migraine and acting as a natural painkiller for acute pain [39,40,41]. Its potent anti-inflammatory properties are due to the significant content of the component parthenolide, a sesquiterpene lactone [13,14,34,38,39,40,41,42]. Its antioxidant, anti-inflammatory, and cytotoxicity activities were investigated, in addition to antibacterial effects [38,39,40,41,42,43].

In the case of *T. corymbosum*, the whole plant or parts of it were reported as remedies for digestive disorders, particularly gastritis, and as antiprotozoal, antibacterial, and antioxidant remedies [43]. Its antimicrobial properties were validated by recent works [14,43].

In this study, we investigated *T. parthenium*, *T. vulgare*, and *T. corymbosum* preserved at the Ghirardi Botanic Garden of the University of Milan (Toscolano Maderno, Brescia, Northern Italy) in the context of a cross-disciplinary project entitled “Botanic Garden factory of molecules…work in progress”, in which the scientific core of the research proposals strictly joined the social perception. We performed: (i) a micromorphological and histochemical investigation on the glandular *indumenta* of leaves and inflorescences; (ii) analyses of the EO compositions obtained from the air-dried, flowered aerial parts collected in June 2021; (iii) an extensive literature search to determine the relationship between the EO profiles and their potential biological and ecological roles. The findings presented herein were made available to the public visiting the garden with the *ad hoc* creation of novel interpretative equipment; in this way, the research results were shared and accessed in an Open Science context, supporting the usability of scientific knowledge for the benefit of society. 

## 2. Results and Discussion

### 2.1. Micromorphological Investigation

#### 2.1.1. Trichome Morphotypes and Distribution Pattern

In each target species, the non-glandular and glandular *indumenta* detected on the examined vegetative and reproductive organs displayed a general uniformity concerning morphotypes and distribution patterns of trichomes in all replicates. 

**Figure 2:**
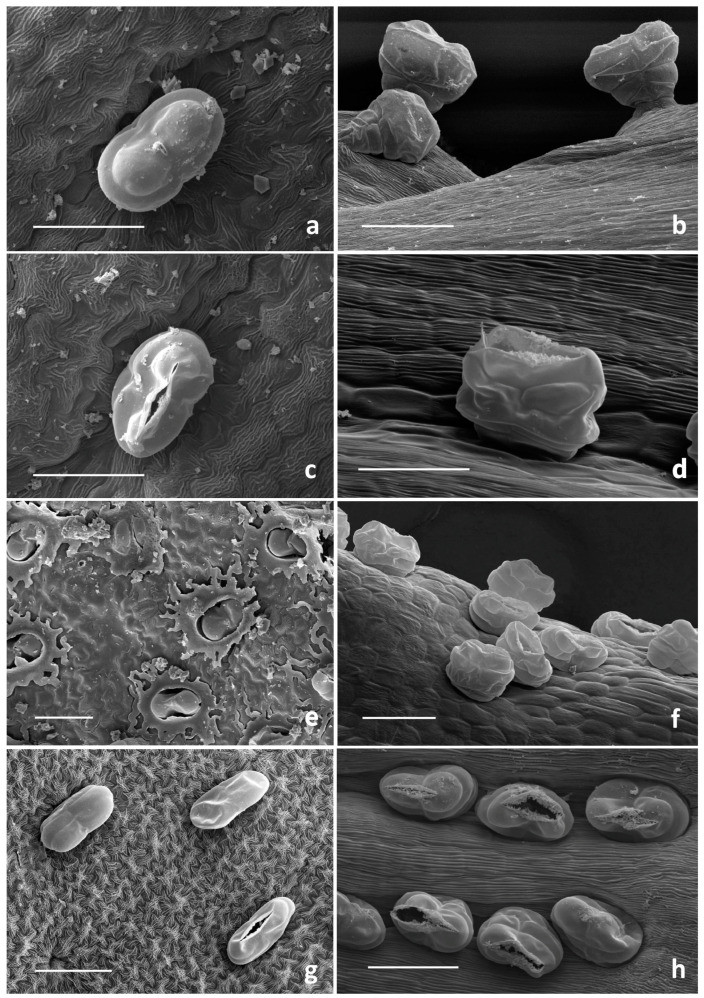
SEM micrographs showing the biseriate glandular trichomes observed on the vegetative and reproductive organs of the examined *Tanacetum* species. (**a**) Sunken biseriate glandular trichome with a small central cuticle opening. (**b**) Protruding biseriate glandular trichomes. (**c**,**d**) Sunken biseriate glandular trichome with the rupture of the cuticle along a line of weakness. (**e**) Sunken glandular trichomes on the leaf abaxial surface with abundant waxy exudates covering the epidermis. (**f**–**h**) Glandular trichome on the abaxial corolla surface of disc florets (**f**)**,** ray florets (**g**) and ovary (**h**). Scale bars: 50 μm.

**Figure 3:**
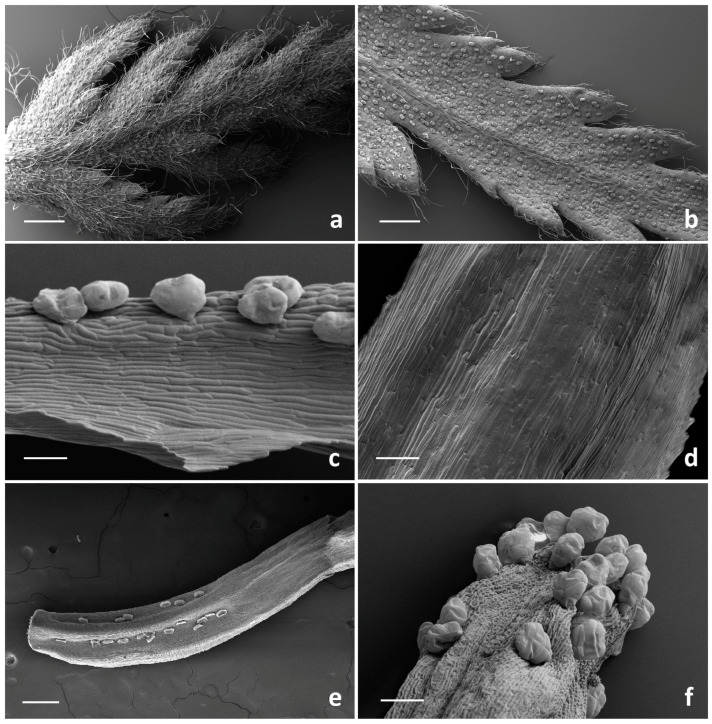
SEM micrographs showing the vegetative and reproductive organs of *Tanacetum vulgare*. (**a**,**b**) General views of the abaxial (**a**) and adaxial (**b**) sides of leaf. (**c**,**d**) Details of the abaxial (**c**) and adaxial (**d**) sides of the involucral bracts. (**e**,**f**) Tubular floret: details of the ovary (**e**) and of the apical portion (**f**) of the corolla. Scale bars: 500 μm (**a**–**c**); 100 μm (**e**); 50 μm (**d**,**f**).

**Figure 4:**
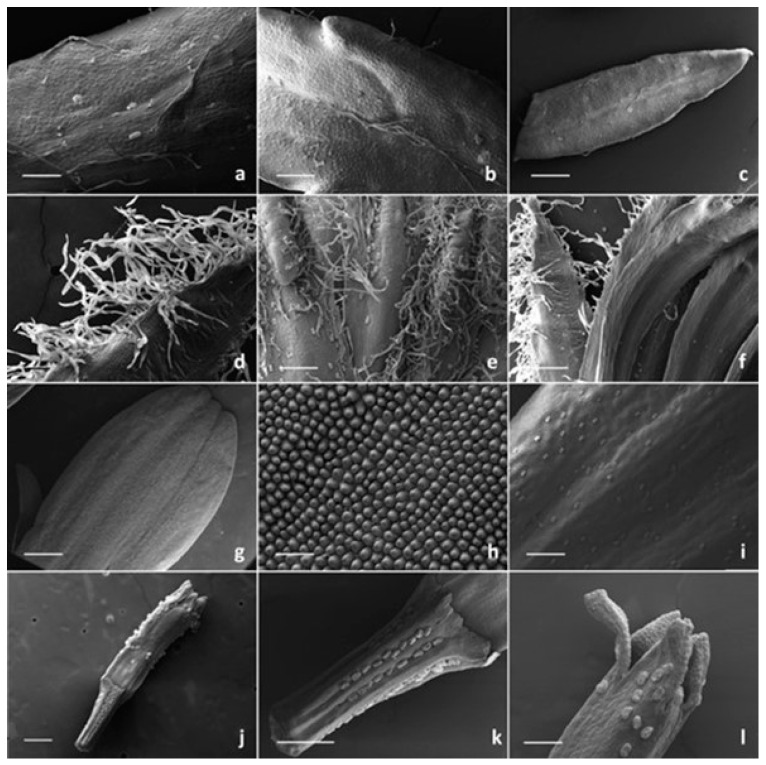
SEM micrographs showing the vegetative and reproductive organs of *Tanacetum parthenium*. (**a**,**b**) Details (**a**,**b**) and general view (**c**) of the abaxial side of the leaf with biseriate glandular trichomes and flagellar hairs. (**d**) General view of the flagellar hairs on the involucral bracts. (**e**–**f**) Details of the abaxial (**e**) and adaxial (**f**) sides of the involucral bracts. (**g**–**i**) Ray floret: details of the adaxial (**g**–**h**) and the abaxial (**i**) sides of the corolla. (**j**–**l**) Ray floret: general view (**j**), details of the ovary (**k**) and the apical portion (**l**) of the corolla. Scale bars: 500 μm (**b**,**c**,**g**); 200 μm (**e**,**f**,**i**–**l**); 100 μm (**d**,**h**).

**Figure 5:**
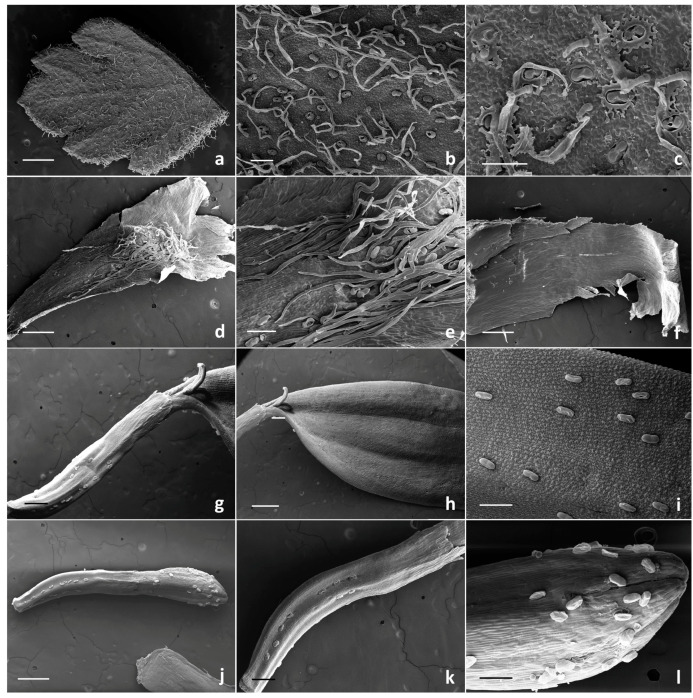
SEM micrographs showing the vegetative and reproductive organs of *Tanacetum corymbosum*. (**a**) General view of the abaxial side of leaf. (**b**,**c**) Details of the abaxial side of the leaf with biseriate glandular trichomes and flagellar hairs. (**d**) General view of the involucral bracts (abaxial side). (**e**) Details of the abaxial side of the involucral bracts with biseriate glandular trichomes and flagellar hairs. (**f**) General view of the involucral bracts (adaxial sides). (**g**–**i**) Ray floret: details of the ovary (**g**), the adaxial (**h**) and the abaxial (**i**) sides of the corolla. (**j**–**l**) Disc floret: general view (**j**), details of the ovary (**k**) and the apical portion (**l**) of the corolla. Scale bars: 500 μm (**a**,**d**,**f**,**h**,**j**); 200 μm (**g**,**k**); 100 μm (**b**,**c**,**e**,**i**,**l**).

SEM investigation on all the examined species allowed us to observe two basic trichome morphotypes: acicular-flagellar non-glandular and ten-celled biseriate glandular (Figure 2, Figure 3, Figure 4 and Figure 5, Table 1). 

The acicular-flagellar non-glandular ones were simple, uniseriate, and consisted of a diverse number of cells, thus resulting as highly variable in length (Figure 3a, Figure 4a–f and Figure 5a–e). However, the exact cell number could not be specified based on the microscopic survey, and the literature data did not report this information for the target species, as well as for other congeneric ones. The basal cells generally protruded from the plant epidermis, and only occasionally appeared swollen. The basal cells were isodiametric, while the distal ones appeared progressively smaller in diameter as they approach the apical tip. They could be more or less stretched, twisted, straight, and oriented at different angles. The above-mentioned features were consistent across the three *Tanacetum* species. 

Their distribution pattern on the examined plant parts is shown in Table 1. 

They were located on both leaf sides, being more abundant on the abaxial one, in all species studied (Figure 3a,b, Figure 4a–c and Figure 5a–c). However, their density was variable. They were more abundant on the leaves of *T. vulgare* and *T. corymbosum*, with a uniform arrangement on the whole lamina (Figure 3a,b), whereas in *T. parthenium,* they were scattered, mainly occurring along the median rib (Figure 4a–c). They also occurred on the abaxial surface of the involucral bracts of *T. parthenium* and *T. corymbosum* (Figure 4e and Figure 5d,e), lacking in *T. vulgare* (Figure 3c). The adaxial side was completely glabrous in all studied species (Figure 3c, Figure 4f and Figure 5f). 

The trichome density appeared greater in *T. parthenium* in comparison to *T. corymbosum*, and they were also more elongated. They showed a peculiar arrangement, being in two rows along either side of the median rib (Figure 4e and Figure 5d,e). Non-glandular trichomes were absent from the florets of the *Tanacetum* species studied (Table 1).

The glandular trichomes were ten-celled and biseriate, composed of two rows of five cells each (Figure 2), as already reported for several species belonging to the family Asteraceae [44]. In the examined organs of the target species, they occurred in various stages of development and consisted of two basal cells mostly placed in the epidermis, one pair of cells forming a stalk and three pairs of cells forming the secretory head (Figure 2). 

The cuticle layer over the secretory head detached and expanded, forming a broad subcuticular cavity, in which the secretory products were stored. LM and SEM observations did not allow us to establish if the storage space overlapped two or three pairs of apical cells. 

The surface of immature glands appeared wrinkled, revealing the close attachment of the cuticle to the secretory upper cell walls. As growth progressed, their surface became smoother since the secretory products were stored within the developing subcuticular cavity formed by the detachment of the cuticle (Figure 2a,b). 

These hairs were generally sunken or slightly protruded from the epidermis (Figure 3, Figure 4 and Figure 5) because of the different elongation of the basal cells. 

They were sunken to various degrees on leaves, involucral bracts, ovaries of both ray and disc florets and on the corolla abaxial side of the tubular florets (Figure 2a,c–h). The hairs on the corolla of the disc ligulate florets were variously protruding (Figure 2b). The secretory products were released externally following the cuticle rupture along a prearranged line of weakness (Figure 2a,d,g,h). Different degrees of cuticle rupture of the mature hairs can be observed: from small central openings, up to increasingly marked longitudinal fractures which sometimes revealed the presence of the secreted materials within the subcuticular cavity (Figure 2a,c,d).

The distribution pattern of the biseriate glandular trichomes is reported in Table 1. Their arrangement on the examined plant parts was consistent among the selected species, while their density showed a high level of variability. In general, glandular trichomes were more abundant on the reproductive organs in comparison to the vegetative ones. On the leaves, the hair density was high on both the leaf sides of *T. vulgare* and *T. corymbosum*, whereas in *T. parthenium,* the biseriate hairs were scattered and less dense on the adaxial surface (Figure 3a,b, Figure 4a–c and Figure 5a–c). 

A peculiar feature emerged from the observation of the leaf abaxial surface of *T. corymbosum*, since it was fully covered by waxy exudates that hid the epidermal appendages, hindering the evaluation of their density. Waxes possessed the appearance of a uniform and continuous film on the organ surface, which was interrupted only at the glandular trichome level. It was possible to observe only the emerging pair of apical secreting cells. The distal cells of the non-glandular trichomes also emerged from the waxy covering (Figure 2e and Figure 5c).

Concerning the involucral bracts of all examined species, the adaxial sides were invariably hairless (Figure 3c, Figure 4f and Figure 5f), and on the adaxial sides, the biseriate trichomes mainly occurred in two rows along either side of the median rib, scattered among the flagellar hairs (Figure 3c, Figure 4e and Figure 5d,e).

The biseriate trichomes were homogeneously distributed on the disc florets and ray florets of *T. corymbosum* and *T. parthenium* (Figure 4g–l and Figure 5g–l) and on the tubular florets of *T. vulgare* (Figure 3e,f).

Trichome density on florets was usually higher on the ovary than on the corolla. On the ovary, they appeared regularly aligned in parallel rows. This regular orientation represents a prelude to the arrangement of these structures on the cypsela surfaces, since they were situated between the ribs in all the investigated *Tanacetum* species [1,2].

On the disc ligulate florets of *T. parthenium* and *T. corymbosum*, the abaxial sides of the ligula was always hairless (Figure 4g–h and Figure 5h), whereas the abaxial ones appeared richly dotted by numerous glands (Figure 4i and Figure 5i). On the corollas, the tubular florets of *T. vulgare*, and on the ray florets of *T. parthenium* and *T. corymbosum*, this kind of trichome was common at the distal portion of the tube (Figure 3f, Figure 4l and Figure 5l). SEM images showed that disc florets had greater trichome density than ray florets in *Tanacetum* (Figure 3, Figure 4 and Figure 5). 

According to our observations, all species examined herein had non-glandular trichomes on the leaves and on the abaxial surfaces of the involucral bracts, while they were absent on the florets. The glandular trichomes were present on all examined plant parts, regardless of their different density ranges in the target species. In the present study, most of the glandular hairs were found on disc and ray florets of all three species. In fact, the glandular trichome density was highest on the florets, followed by leaves and involucral bracts. This evidence is in accordance with the observations on several *Tanacetum* species by Majdi et al. [31]. The detected elements of variability concerned the much higher and the much lower trichome density on the leaves of *T. vulgare* and *T. parthenium*, respectively, in comparison to the other analyzed species; the occurrence of waxy exudates covering the leaf abaxial sides of *T. corymbosum*; and the absence of flagellar trichomes on the abaxial side of the involucral bracts in *T. vulgare*. These differences in the trichome features could help to differentiate the selected *Tanacetum* species; however, a greater sampling effort will be needed to establish their actual diagnostic value. The taxonomic significance of trichomes in the identification of some members of the Asteraceae was reported by several authors [31,45,46].

Few micromorphological contributions were found for the target species. Flagellar covering and biseriate glandular trichomes were observed on both vegetative and reproductive organs by light and scanning electron microscopy in *T. vulgare* and *T. parthenium* [30,31,32,33]. The morphological features of the covering hairs, as well as biseriate trichomes, were uniform with literature data, regardless of their localization on the different plant parts and their density. The element of novelty lies in the information on the *indumentum* of *T. corymbosum*, reported for the first time herein.

#### 2.1.2. Histochemistry of the Biseriate Glands 

**Figure 6:**
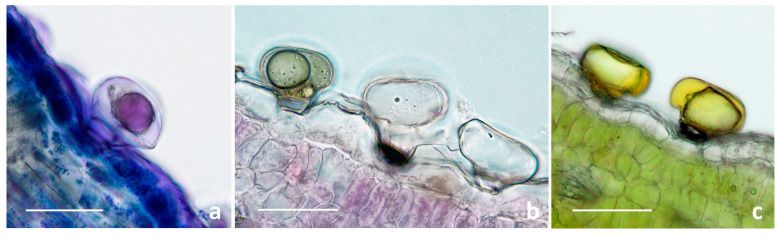
LM micrographs showing the histochemical results on the secretory materials of the biseriate glandular trichomes of the selected *Tanacetum* species. (**a**) Nadi reagent. (**b**) Ruthenium Red test. (**c**) Ferric Trichloride test. Scale bars: 50 μm.

To define the histochemical profiles of the secretory products of the biseriate glands, we applied specific dyes. The overall results proved the chemical consistency of the production in secondary metabolites in all the target species, regardless of the distribution pattern on the vegetative and reproductive organs (Table 2; Figure 6).

Our findings proved the production and release of lipophilic substances, considering the positive responses for total lipids, neutral lipids, and terpenoids (Table 2). The latter, represented as dark violet small droplets or larger clusters, were localized in the secretory cells and subcuticular cavity (Figure 6a). Drops of secretions localized in the secretory cells emitted intense primary fluorescence under UV excitation. 

Polysaccharides were only sporadically evidenced in the secretory cells and subcuticular cavities, by weak light-blue or pink–red coloration to the hydrophilic dyes Alcian Blue and Ruthenium Red, respectively. However, these glands remained generally colorless (Table 2; Figure 6b). The samples stained with FeCl_3_ (Table 2; Figure 6c) and with AlCl_3_ and Naturstoff reagent A (Table 2) exhibited invariably positive responses, indicating the copious synthesis of polyphenols and, in particular, flavonoids. 

The secretion of terpenoids by biseriate glandular hairs was also shown in the leaf of *T. vulgare* grown in vitro [33]. Our results also revealed the productivity of major polyphenolic fractions, whereas Devrnja et al. [33] documented a negative response following the application of FeCl_3_. Moreover, polysaccharides were also reported by these authors, whereas they were only sporadically evidenced herein.

### 2.2. Phytochemical Investigation

The chemical compositions of the EOs from the flowered aerial plant parts of the selected *Tanacetum* species are reported in Table 3 and Table S1. 

The obtained oil yields ranged from 0.05% in *T. corymbosum* and 0.09% in *T. parthenium* up to 0.35% in *T. vulgare*. The most complex EO profile, due to the presence of the highest number of total compounds, was the one obtained from *T. vulgare* (67 components), followed by *T. parthenium* (28) and *T. corymbosum* (17). 

In *T. vulgare* (*Tv*) EOs, the dominant compound classes were oxygenated sesquiterpenes (30.15%) and oxygenated monoterpenes (22.70%), followed by sesquiterpene hydrocarbons (17.38%). The dominant compounds were: caryophylladienol (no. 67 in Table 3, 5.92%), farnesol (74, 5.92%), α-calacorene (61, 4.92%), *p*-cymene (15, 3.37%), and cyperene (48, 3.22%). 

The *T. parthenium* (*Tp*) EOs were dominated by oxygenated monoterpenes (61.51%) and oxygenated sesquiterpenes (33.72%). The most abundant compounds were camphor (29, 56.83%) and farnesol (74, 28.83%). The six exclusive compounds were: 2-hexyn-1-ol (2, 0.21%), 1-hexanol (4, 0.27%), 2,2,6-trimethyl-3-keto-6-vinyltetrahydropyran (24, 0.61%), α-pinocarvone (31, 0.22%), *cis*-geraniol (37, 0.90%), and safrole (44, 0.25%).

The *T. corymbosum* (*Tc*) EO profile displayed oxygenated monoterpenes (53.01%), oxygenated sesquiterpenes (27.88%), and non-terpenic derivatives (16.32%) as the main chemical classes. The principal compounds were camphor (29, 49.36%), *α*-santalone (82, 21.58%), and hexanal (1, 11.0%). Six compounds were exclusive: α-santalone (82), artemisia alcohol (19, 0.66%), silphiperfol-5-ene (45, 0.74%), *trans*-verbenyl isovalerate (53, 0.84%), shyobunol (78, 1.92%), and thujopsenal (77, 2.23%). 

The EO profiles investigated herein displayed a high qualitative variability in terms of total number of compounds, dominant chemical classes, main compounds, and exclusive constituents. 

The three investigated EO profiles had six common compounds, i.e., hexanal (1), 2-hexenal (3), 1,8-cineole (14), camphor (29), α-santalol (70) and costol (75), generally exhibiting a high level of quantitative variability. Costol and α-santalol displayed comparable patterns of variability in the percentage values across the investigated samples, ranging from 0.58% up to 1.15% and from 0.50% up to 0.98%, respectively. The relative amounts of 1,8-cineole were consistent in *Tv* and *Tc* EOs (1.35% and 1.49%, respectively), whereas they were significantly lower in *Tp* EOs (0.13%); a divergent trend resulted for hexanal and 2-hexenal, with lower percentages in *Tv* and *Tp* EOs in comparison to *Tc* EOs, where these two compounds were among the dominant ones in the profiles. Finally, camphor was the main constituent of *Tp* and *Tc* EOs, with percentages of 56.83% and 49.36%, respectively, whereas in *Tv* EOs, it was a minor compound. In addition to the ubiquitous molecules, *Tv* and *Tp* EOs shared 12 further constituents, among which farnesol (74, 5.92% and 28.83%) was one of the dominant compounds in both the profiles; *Tv* and *Tc* EOs were characterized by three further common constituents, i.e., 2,5,5-trimethyl-3,6-heptadien-2-ol (13), 6-isopropyl-3-methyl-7-oxabicyclo [4.1.0]-heptan-2-one (40) and γ-elemene (57), each present in comparable amounts in the profiles; *Tp* and *Tc* EOs shared only one further compound, i.e., isopinocarveol (32, 0.93% and 0.30%).

Comparing the taxonomic treatment at the section level (*T. vulgare* belonging to *Tanacetum* sect. *Tanacetum* and *T. parthenium* and *T. corymbosum* included within *Tanacetum* sect. *Pyrethrum*) and the quali-quantitative features of the obtained EO compositions, we note that the phytochemical profiles, besides the morphological features, seemed to support the infrageneric classification: (a) *T. vulgare* showed the more complex profile, with a huge number of exclusive compounds, in comparison to the other investigated samples and with oxygenated sesquiterpenes as the main chemical class; (b) the inclusion of *T. parthenium* and *T. corymbosum* within the same section appears consistent with the occurrence of oxygenated monoterpenes as the main compound class and by the presence of camphor as the dominant constituent of the EO profiles. However, a lack of consistency emerges if we consider the number of additional common compounds in the profile (*Tp* and *Tc* EOs with only one common compound versus *Tv* and *Tp* EOs and *Tv* and *Tc* EOs sharing 12 and 3 common further constituents, respectively).

The scientific literature on the characterization of tansy EO composition is rich and includes samples from different European countries (Italy, Serbia, Romania, Poland, Slovakia, Norway, Estonia, Lithuania) and from Canada ([13] and literature therein). The whole aerial parts harvested at the full blooming stage were the most studied plant material, although leaves and inflorescences, suitably separated, were also the target of study for Lithuanian samples. The drying process was the most common conservation procedure. The oil yields, as in the samples analyzed herein, varied between 0.1 and 0.5%, but in some cases, it was reported up to 1.9% [13,47,48].

The *Tv* EO profile obtained herein was unusual, as the literature data generally report fewer complex profiles with two or three major compounds at percentages above 10.0%; the dominant compounds identified in the previously investigated samples were β-thujone, trans-thujone, chrysanthenyl acetate, camphor, and 1,8-cineole [38]. The first three components did not occur in our samples, while camphor and 1,8-cineole accounted for relative amounts in the range 1.0–2.0%.

The chemotypic panorama was wide and diversified. As an example, more than 15 different chemotypes of tansy from Scandinavia and the Baltic region were described. In Lithuania, four groups were defined: 1,8-cineole, trans-thujone, camphor, and myrtenol [49], the latter compound occurring in our sample with a percentage of 0.97% [50]. In Norway, seven chemotypes were identified: α-thujone, β-thujone, camphor, chrysanthenyl acetate/chrysanthenol, chrysanthenone, artemisia alcohol, and 1,8-cineole [48]. In our samples, the chrysanthenol derivatives and artemisia alcohol were lacking.

According to previous research, factors that can impact the chemical polymorphism of tansy EOs include the following: pedoclimatic conditions, habitat type, geographical origin, plant material used, wild-growing or cultivated plants, and the existence of different genotypes or chemotypes [48,49,51].

The profiles examined herein, along with those obtained for Sicilian samples [51], showed the most complex compositions compared to the other EOs known from the literature. In fact, our EO profile showed invariably the greatest number of several minor compounds.

Concerning the comparison with the previously investigated Italian samples, our study represents the first survey performed on *T. vulgare* in northern Italy. Formisano et al. [51] described *T. vulgare* subsp. *siculum* as a thujone chemotype, suggesting that the high thujone content could be explained by the high sun exposure of plants [48].

The high qualitative complexity of the tansy EO profile did not allow us to associate the plants we investigated to a specified chemotype.

Concerning feverfew, *T. parthenium* EOs were the subject of several previous works on samples from different geographical areas (England, the Netherlands, Kosovo, Turkey, Egypt, Iran, Tajikistan) [13,14,34,38,39,40,41,42,43,52]. The comparison with literature data proved that camphor (29) was invariably the dominant component with percentages in the range of 43.0–60.0% of the total components identified [52]. Other main compounds detected in variable relative amounts in the previously investigated *T. parthenium* EOs were camphene and chrysanthenyl acetate [52], which were lacking in our samples.

*Tp* EOs examined herein also showed nine other compounds common to profiles presented in literature: hexanal (1), 1,8-cineole (14), γ-terpinene (17), *cis*-sabinene hydrate (18), nonanal (23), *p*-meth-2-en-1-ol (28), endo-borneol (33), terpinen-4-ol (34), and caryophylladienol (67). All these compounds were reported in moderate amounts or in traces, with a high quantitative variability across samples. The variations in the chemical composition of *T. parthenium* EOs of different origin may be due to exogenous and endogenous factors, such as climate, developmental stages, soil type, and cultivated and wild-growing plants [52]. The second major compound found in our *Tp* EOs, farnesol (74), was not previously reported for *T. parthenium*, but is a minor constituent common to the *T. vulgare* profile. Farnesol has been reported as a molecule inducing programmed cell death in fungi [53], and its synthesis might be downregulated in more arid habitats where the presence of fungal spores is less frequent with respect to more humid areas. *T. corymbosum* is commonly observed in arid environments in Europe [54], while *T. parthenium* and *T. vulgare* have a wide distribution including more humid environments [54].

Considering *Tc* EOs, the profile obtained herein was very different from those reported in the literature [54,55]. In our samples, the main chemical classes were oxygenated mono- and sesquiterpenes, whereas the EO profiles in the literature were typified by mono- and sesquiterpenoids. Furthermore, the main components in our samples were camphor (29), *α*-santalone (82), hexanal (1), and 2-hexenal (3), while we highlighted the noteworthy absence of cadinene derivatives, which were the dominant constituents of EOs obtained by Thomas [54], and of germacrene D and *β*-farnesene, main compounds in Moldavian profiles [50].

Regarding the literature data on the biological activities documented for the essential oils of the target species, tansy EOs exhibit antioxidant, antihypertensive, and diuretic effects, as well as antibacterial, antifungal, anthelmintic, and acaricidal activities [13,14,35,36,37]. For feverfew EOs, antioxidant, anti-inflammatory, and cytotoxicity activities were investigated, in addition to antibacterial effects [38,39,40,41,42,43]. For corymbflower tansy, antimicrobial properties were validated by recent works [14,43].

Since our EO profiles were divergent from those reported in the literature, we evaluated the potential biological activity, along with their ecological roles, of the main compounds: camphor (29), α-santalone (82), hexanal (1), 2-hexenal (3), farnesol (74), α-calacorene (61), and caryophylladienol (67).

In terms of biological activity, camphor (29), the main component in *Tp* and *Tc* EOs, is used to relieve inflammation deriving from rheumatism, joint and muscle pain, and bronchitis due to its analgesic properties [55].

Farnesol (74), the second main compound in *Tv* and *Tp* EOs, possesses antioxidant effects, along with various pharmacological activities, such as anticancer, and it is used in cosmetics to emphasize the fragrance of floral perfumes [56,57].

No direct studies on the biological activity were reported for hexanal (1) and 2-hexenal (3), both among the main compounds in *Tc* EOs, nor for α-calacorene (61), one of the main components of *Tv* EOs. No information was available for α-santalone (82), the second main compound in *Tc* EOs, and for caryophylladienol (67), one of the principal compounds in *Tv* EOs.

Regarding the ecological roles of the above-mentioned compounds, camphor (29) showed antibacterial and antifungal properties [58,59], and it was studied for its repellent and insecticidal properties against different species of beetles [60]. Similarly, hexanal (1) and 2-hexenal (3) showed preeminent antimicrobial activity due to their aptitude to increase membrane permeability and cell death in different post-harvest pathogens [61,62], so that they are widely studied for potential applications in food quality preservation [63,64]; these two compounds were also recognized in the pheromone formation process of several insect species [65]. Farnesol, which was recognized as a potent attractant for mites, is used combined with specific biocides for mite control in food crops [66].

The compounds α-calacorene (61) was documented to have antimicrobial activity against various Gram-negative bacteria and fungi [67]. Meanwhile, information on the ecological potential of α-santalone (82) and caryophylladienol (67) is lacking.

### 2.3. Scientific Dissemination

The scientific data described in the “Micromorphological investigation” and “Phytochemical investigation” sections were joined in the design and realization of original interpretative and iconographic apparatuses for the target species at the Ghirardi Botanic Garden (Toscolano Maderno, Brescia, Italy). Noteworthy is also the inclusion of original line drawings, specially created, which highlight the macroscopic diagnostic characteristics of the target plant.

**Figure 7:**
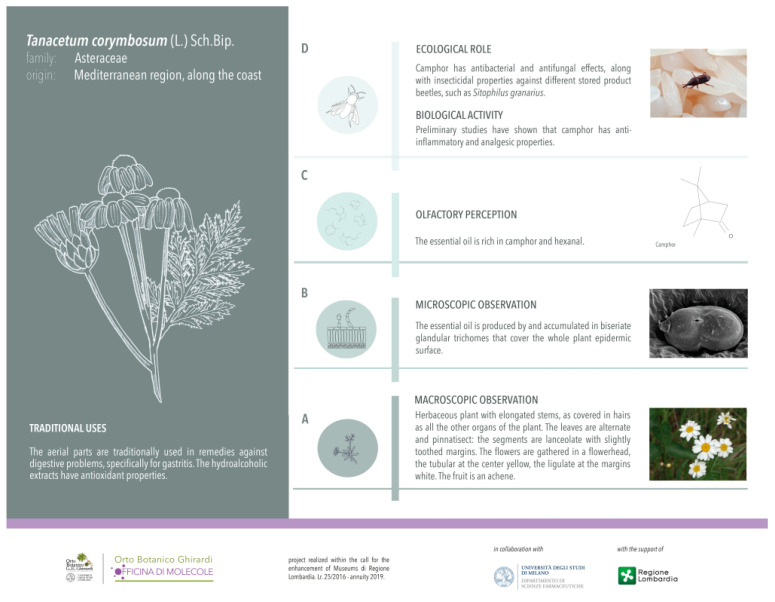
New labeling of *Tanacetum corymbosum* (L.) Sch.Bip. at the Ghirardi Botanic Garden (University of Milan, Toscolano Maderno, Brescia, Italy). The information on the scientific results on the target species are reported according to the four-scale research levels of the main project “Ghirardi Botanic Garden, factory of molecules...work in progress”: (**A**) macroscopic observation, through the description of diagnostic macromorphological features. (**B**) microscopic observation, referring to the sites secreting the secondary metabolites. (**C**) olfactive perception, through the phytochemical characterization of their profile. (**D**) bio-ecological, regarding the evaluation of their biological activity and ecological significance.

In addition to the plant macroscopic features, the micromorphology of the secretory structures, the main components of the EO profile, along with data concerning their ecological roles and biological activity were highlighted (Figure 7).

## 3. Materials and Methods

### 3.1. Plant Material

*Tanacetum vulgare* L., *Tanacetum parthenium* (L.) Sch.Bip., and *Tanacetum corymbosum* (L.) Sch.Bip. are cultivated at the Ghirardi Botanic Garden, Department of Pharmaceutical Sciences, University of Milan (Toscolano Maderno, BS). Prof. G. Fico and Prof. C. Giuliani identified the plants according to Pignatti et al. [27].

Each investigated target species is present at the study site as single individuals. The analyzed plant material, therefore, derived from the same specimen of which several ramets (with inflorescences at the same phenological phase of anthesis) were gathered. The samplings were performed during the blooming period in June 2021.

Voucher specimens of *T. vulgare*, *T. parthenium,* and *T. corymbosum*, were labeled with the codes GBG2021/110, GBG 2021/111, and GBG2021/112, respectively, and deposited in the Herbarium of the Ghirardi Botanic Garden.

### 3.2. Chemicals

Solvents were of gradient-grade purity and purchased from either Exacta Optech Labcenter SpA (San Prospero, Italy) or VWR International (Milan, Italy). All reagents were of reagent-grade purity, purchased from Sigma Aldrich (Merck group, Milan, Italy), Fisher Scientific Italy (Rodano, Italy), or VWR International (Milan, Italy), and used as received.

### 3.3. Micromorphological Investigation

We described the structure, the distribution pattern, and the histochemistry of trichomes on the vegetative and reproductive organs by means of scanning electron microscopy (SEM), light microscopy (LM), and fluorescence microscopy (FM). In each of the selected species and for each examined plant part, at least ten replicates were analyzed to evaluate the variability level of the micromorphological features.

#### 3.3.1. Scanning Electron Microscopy (SEM)

Leaves, involucral bracts, disc florets, and ray florets were hand-prepared, fixed in FAA solution (formaldehyde:acetic acid:ethanol 70% = 5:5:90) for 24 h, dehydrated in an ascending ethanol series up to absolute, and critical-point dried. The samples were mounted on aluminum stubs and gold-coated. Observations were performed under a Zeiss^®^ EVO MA15 SEM operating at 10 kV at the Interdepartmental Center for Electron Microscopy and Microanalysis Services (M.E.M.A.) of the University of Florence (Florence, Italy).

#### 3.3.2. Light Microscopy and Fluorescence Microscopy

The micromorphological survey under light microscopy and fluorescence microcopy was performed on the vegetative and reproductive organs from the same individual. We used both fresh material and fixed samples included in historesin (Technovit^®^ 7100, Heraeus Kulzer GmbH & Co. KG, Wehrheim, Germany).

For the fresh material, sections ranging from 30 to 50 µm in thickness were obtained using a vibratome and/or a cryostat.

Samples were also fixed in FAA solution for 48 h at 4 °C. Subsequently, fixed samples were washed in 70% ethanol for 24 h; they were then dehydrated progressively by treatment with 80% ethanol for 2 h, 95% ethanol for 2 h, and then twice in absolute ethanol for 2 h each. Pre-inclusion was then performed first with ethanol and historesin in 1:1 ratio for one night, then with 1:2 ratio for 2 h, and in pure historesin for 3 h. Finally, the inclusion was done in a polypropylene capsule with addition of hardener in ratio 1:15 of basic resin. The historesin samples were cut in 2 µm sections with an ultramicrotome.

The following dyes were used [46]: Toluidine Blue as a general staining; Sudan III/IV and Fluoral Yellow-88 for total lipids; Nile Red for neutral lipids; Nadi reagent for terpenes; Periodic Acid-Schiff (PAS) reagent for total polysaccharides; Alcian Blue for mucopolysaccharides; Ruthenium Red for pectins; Ferric Trichloride for polyphenols; Aluminum Trichloride and Naturstoff reagent A for flavonoids. Control procedures were carried out concurrently. Observations were made with a Leitz DM-RB Fluo optical microscope equipped with a Nikon digital camera.

### 3.4. Phytochemical Investigation

#### 3.4.1. Preparation of Essential Oils (EOs)

The plant material was air-dried at room temperature and away from direct light sources for 10 days.

For each target species, the samples were weighed, ground, put into 4 L flasks filled with deionized water, with a 1:10 plant material/water ratio, and subjected to hydrodistillation using a Clevenger-type apparatus for 3 h, checking that after this time the volume of the obtained oil remained constant. Once obtained, the essential oil was decanted and separated from water, of which residual drops were removed using anhydrous sodium sulfate. The oil yield was estimated on a dry weight basis (*w*/*w*).

Due to the presence of only one individual for each selected species at the Ghirardi Botanic Garden, no replicate was performed for distillation. Three replicas were per-formed in oil dilution for GC-MS analysis to evaluate variation in essential oil composition; results showed only small variations in relative amounts of single components, which did not affect percentage data reported in Table 3 and Table S1.

Given that the target species were present as single individuals, this did not allow us to perform replications in sampling; however, replications were performed in the preparation of the essential oil dilutions, to assess the variability in the analytical determination of individual compounds.

#### 3.4.2. GC-MS Analysis of Essential Oils

EOs were analyzed by GC-MS using a Thermo Scientific TRACE ISQ QD Single Quadrupole GC-MS (Thermo Fisher Scientific, Waltham, MA, USA). EO separation was performed by a capillary column VF-5ms (5% phenyl-methyl-polisiloxane, length 30 m, 0.25 mm i.d., 0.1 μm film thickness); the temperature gradient was: 8 min at 50 °C, then 4 °C/min up to 60 °C, then 6 °C/min from 60 °C to 160 °C, and finally 20 °C/min from 160 °C to 280 °C. Injector and detector temperatures were set to 280 °C; carrier gas was He, flux was 1 mL/min, and the mass range detected was 50–500 m/z. EOs were analyzed diluted 1:50 with n-hexane, with an injection volume of 1 µL.

Mass spectra were analyzed by Wiley Mass spectra Library, NIST Mass Spectral Search Program e NIST Tandem Mass Spectral library 2.3; compounds were identified by mass fragmentation and comparison of retention indices, calculated using a C8–C30 series of *n*-alkanes (Sigma-Aldrich, Milan, Italy), with data stored in mass databases (NIST 18, Wiley, Hoboken, NJ, USA), Adams [68]. Percentage values of essential oil components were obtained from the peak areas in the chromatogram without the use of correction factors.

## 4. Conclusions

This multidisciplinary work on the three target species of *Tanacetum* supported the following considerations.

(i). The combination of digital light microscopy and scanning electron microscopy enabled us to deepen the knowledge about the non-glandular and glandular *indumenta* on the vegetative and reproductive organs; specifically, our investigation proved a great affinity regarding the trichome morphotypes, their distribution pattern and density across the studied taxa, along with a similar histochemical profiles of the secretory products (mainly terpenes and flavonoids) of the glandular hairs; specifically, the overall information on *Tanacetum corymbosum* represented an element of novelty in the panorama of the current literature.
(ii). The phytochemical characterization explained the variability of the EO profiles, when compared with the literature data. We addressed our work on the investigation of species cultivated at the same study site and collected in the same period with the special intent to make an internal comparison, limiting every single variable. The study revealed great complexity in the profile of the EOs obtained from the flowering aerial parts in the case of *T. vulgare*, compared to *T. parthenium* and *T. corymbosum*: the latter appeared to represent an established source of compounds such as camphor, farnesol, or α-santalone.
(iii). The phytochemical diversity, particularly in relation to the major compounds present, may reflect the diversified use of these three species in relation to the interest versus a peculiar biological activity.
(iv). Although the composition of the volatile organic fraction was not investigated, based on the literature data, we assumed that the main EO components exert ecological roles related to a dominant defense action in all the studied species. Specifically, they include repulsive agents against plant pathogens such as microbes, beetles, and mites.
(v). The scientific results were channeled into the creation of an original interpretative apparatus for the target species at the Ghirardi Botanic Garden, giving rise to a direct exchange of information with the public, immediately following the research, thus emphasizing the concept of Open Science policy.

## Figures and Tables

**Table 1:** Distribution pattern of the flagellar non-glandular and biseriate glandular trichomes on the examined vegetative and reproductive organs of the selected *Tanacetum* species.

Trichomes Type	Species	Leaf		Involucral Bract		Ligulate Floret		Tubular Floret	
		adax.	abax.	adax.	abax.	ovary	corolla	ovary	corolla
**Flagellar** **non-glandular**	***T. vulgare***	++	+++	−	−			−	−
	***T. parthenium***	±	+	−	++	−	−	−	−
	***T. corymbosum***	+	++	−	++	−	−	−	−
**Ten-celled biseriate** **glandular**	***T. parthenium***	+	+	−	++	++	++	+++	++
	***T. vulgare***	++	+++	−	++			++	++
	***T. corymbosum***	++	+++	−	++	++	++	++	++

Symbols: (–) missing, (±) sporadic, (+, ++, +++) increasing presence of trichomes.

**Table 2:** Results of the histochemical tests on the biseriate glands of the vegetative and reproductive organs of the selected *Tanacetum* species.

Stains	Target Compounds	*T. vulgare*	*T. parthenium*	*T. corymbosum*
**Fluoral Yellow-088**	Total lipids	++	+	+
**Nile Red**	Neutral lipids	+	+	+
**Nadi reagent**	Terpenoids	++	++	++
**PAS reagent**	Total polysaccharides	−	+	−
**Ruthenium Red**	Acid polysaccharides	−	±	−
**Alcian Blue**	Muco-polysaccharides	−	−	±
**FeCl_3_**	Polyphenols	++	++	++
**AlCl_3_**	Flavonoids	+	+	+
**Naturstoff reagent A**	Flavonols	+	+	+

Symbols: (−) negative response; (±) faintly positive response; (+) positive response; (++) intensely positive response.

**Table 3:** GC-MS profiles of the essential oils obtained from the flowered aerial parts of the selected *Tanacetum* species collected in June 2021. The common compounds are highlighted in grey color. *Tv* = *T. vulgare*; *Tp* = *T. parthenium; Tc* = *T. corymbosum*.

					Relative Abundance (%)		
No.	LRI^a^	LRI^b^	Type	Compound	*Tv*	*Tp*	*Tc*
1	769	776	NH	hexanal	0.59	0.55	11.00
2	821	821	NH	2-hexyn-1-ol	-	0.21	-
3	843	835	NH	2-hexenal	0.25	0.41	4.57
4	863	854	MH	1-hexanol	-	0.27	-
5	869	868	NH	1,6-dimethylcyclohexene	0.26	-	-
6	897	902	MH	santolina triene	2.48	-	-
7	922	926	NH	2,5,5-trimethyl-1,3,6-heptatriene	0.82	-	-
8	926	925	MH	3-thujene	0.36	-	-
9	937	933	MH	α-pinene	1.11	-	-
10	955	946	MH	camphene	1.95	-	-
11	981	973	MH	β-pinene	0.82	-	-
12	991	1017	MO	myroxide	1.57	-	-
13	1004	1000	MH	2,5,5-trimethyl-3,6-heptadien-2-ol	0.43	-	0.47
14	1037	1022	MO	1,8-cineole	1.35	0.13	1.49
15	1037	1025	NH	*p*-cymene	3.37	-	-
16	1062	1072	NH	hotrienol	2.51	-	-
17	1067	1060	MH	γ-terpinene	0.29	0.31	-
18	1075	1070	MH	*cis*-sabinene hydrate	2.38	0.53	-
19	1076	1072	MO	artemisia alcohol	-	-	0.66
20	1079	1079	MH	terpinolene	0.18	-	-
21	1086	1086	MO	linalool	1.53	-	-
22	1091	1074	MH	*p*-cymenene	-	-	1.54
23	1103	1083	NH	nonanal	0.97	0.15	-
24	1106	1108	NH	2,2,6-trimethyl-3-keto-6-vinyltetrahydropyran	-	0.61	-
25	1107	1167	NH	2-nonen-1-ol	0.65	0.72	-
26	1115	1207	MO	carveol	2.05	-	-
27	1128	1105	MO	fenchol	0.46	0.27	-
28	1132	1126	MO	*p*-menth-2-en-1-ol	1.79	0.33	-
29	1156	1146	MO	camphor	1.80	56.83	49.36
30	1170	1181	MO	myrtenol	0.97	-	-
31	1146	1164	MO	α-pinocarvone	-	0.22	-
32	1150	1180	MO	isopinocarveol	-	0.93	0.30
33	1182	1167	MO	endo-borneol	2.23	1.11	-
34	1189	1182	MO	terpinen-4-ol	0.66	0.78	-
35	1205	1171	MO	myrtenal	1.92	-	-
36	1215	1192	MO	(*Z*)-piperitol	0.18	-	-
37	1233	1237	MO	*cis*-geraniol	-	0.90	-
38	1238	1305	MO	myrtenyl acetate	1.81	-	-
39	1246	1260	MO	lyratyl acetate	0.30	-	-
40	1265	1261	MO	6-isopropyl-3-methyl-7-oxabicyclo [4.1.0]-heptan-2-one	1.05	-	1.20
41	1274	1250	MO	geranyl vinyl ether	0.32	-	-
42	1281	1272	MO	*p*-mentha-1,8-dien-3-one	0.12	-	-
43	1288	1285	MO	bornyl acetate	1.28	-	-
44	1289	1287	NH	safrole	-	0.25	-
45	1307	1331	NH	silphiperfol-5-ene	-	-	0.74
46	1319	1350	MO	α-terpinyl acetate	0.27	-	-
47	1353	1398	MO	β-elemene	1.06	-	-
48	1363	1399	SH	cyperene	3.22	-	-
49	1378	1376	SH	α-copaene	0.22	-	-
50	1392	1398	SH	ciclohexane, 1-ethenyl-1-methyl-2,4-bis(1-methylethenyl)	2.13	-	-
51	1426	1419	SH	caryophyllene	1.99	-	-
52	1462	1496	SH	*cis*-α-bisabolene	0.53	-	-
53	1470	1510	SO	*trans*-verbenyl isovalerate	-	-	0.84
54	1482	1483	SH	α-curcumene	0.95	-	-
55	1496	1499	SH	eremophilia-1(10),11-diene	1.62	-	-
56	1502	1405	SH	longifolene	0.55	-	-
57	1516	1433	SH	γ-elemene	0.61	-	0.79
58	1523	1524	SH	δ-cadinene	0.63	-	-
59	1539	1532	SO	cubenene	0.11	-	-
60	1546	1576	SO	spathulenol	0.45	-	-
61	1549	1542	SH	α-calacorene	4.92	0.27	-
62	1551	1581	SO	caryophyllene oxide	-	-	-
63	1570	1572	SO	8-acetoxycarvo-tanacetone	2.08	-	-
64	1579	1586	SO	ledol	2.05	-	-
65	1595	1632	SO	longiverbenone	2.00	0.44	-
66	1622	1606	SO	humulene epoxide	0.29	-	-
67	1642	1637	SO	caryophylladienol	5.92	2.19	-
68	1654	1638	SO	isospathulenol	0.86	-	-
69	1665	1672	SO	aromadendrene oxide (I)	1.30	-	-
70	1672	1681	SO	α-santalol	0.79	0.98	0.50
71	1688	1694	SO	β-santalol	1.36	-	-
72	1707	1693	SO	germacra-3,7(11),9-trien-6-one	0.44	-	-
73	1716	1678	SO	aromadendrene oxide (II)	1.43	-	-
74	1757	1713	SO	farnesol	5.92	28.83	-
75	1783	1778	SO	costol	1.15	0.58	0.80
76	1788	1701	SO	shyobunol	-	-	1.92
77	1790	1724	SO	thujopsenal	-	-	2.23
78	1795	1695	SO	7-isopropyl-4,10-dimethylenecyclodec-5-enol	1.39	-	-
79	1822	1763	SO	*cis*-lanceol	0.36	0.43	-
80	1838	1777	SO	15-hydroxy-α-muurolene	0.34	-	-
81	1853	1844	SO	hexahydrofarnesyl acetone	1.89	-	-
82	1869	1867	SO	α-santalone	-	-	21.58
				Oil Yield (%)	0.35	0.09	0.05
				Total Identified	89.67	100.00	100.00
				Non-terpenic derivatives (NH)	9.42	3.18	16.32
				Monoterpene hydrocarbons (MH)	10.02	1.32	2.01
				Oxygenated monoterpenes (MO)	22.70	61.51	53.01
				Sesquiterpene hydrocarbons (SH)	17.38	0.27	0.79
				Oxygenated sesquiterpenes (SO)	30.15	33.72	27.88

The main common compounds are highlighted in grey. LRI^a^ = Linear Retention Index, experimentally obtained on a VF-5MS column using a C_7_–C_30_ mixture of *n*-alkanes. LRI^b^ = Linear Retention Index as reported in NIST databases.

## Data Availability

The original contributions presented in the study are included in the article/Supplementary Materials, further inquiries can be directed to the corresponding author.

## Supplementary Materials

The following supporting information can be downloaded at: https://www.mdpi.com/article/10.3390/plants13020155/s1, Table S1: GC-MS profiles of the essential oils obtained from the flowered aerial parts of the selected *Tanacetum* species collected in June 2021. The individual compounds are grouped according to different chemical classes and the IUPAC names are reported. The compounds common to the three target species are indicated in grey color.
