# Supplementary material for: An Integrative Approach to Selected Species of Tanacetum L. (Asteraceae): Insights into Morphology and Phytochemistry
Source: Plants (Basel). 2024 Jan 5;13(2):155. doi: 10.3390/plants13020155 (PMC10819483; doi:10.3390/plants13020155)
Supplement: Supplementary file 1 [file plants-13-00155-s001.zip › Table S1.pdf]

Table S1. GC-MS profiles of the essential oils obtained from the flowered aerial parts of the selected *Tanacetum* species collected in June 2021. The individual compounds are grouped according to different chemical classes and the IUPAC names are reported. The common compounds to the three target species are indicated in grey color.
Tv = T. vulgare; Tp = T. parthenium; Tc = T. corymbosum.

N.	LRI[a]	LRI[b]	Compound	IUPAC name	Relative abundance (%) *Tv*	*Tp*	*Tc*
Non terpenic hydrocarbons and derivatives:							
**1**	769	776	hexanal	hexanal	0.59	0.55	11.00
**2**	821	821	2-hexyn-1-ol	2-hexyn-1-ol	-	0.21	-
**3**	843	835	2-hexenal	2-hexenal	0.25	0.41	4.57
**5**	869	868	1,6-dimethylcyclohexene	1,6-dimethylcyclohexene	0.26	-	-
**7**	922	926	2,5,5-trimethyl-1,3,6-heptatriene	2,5,5-trimethyl-1,3,6-heptatriene	0.82	-	-
**4**	863	854	1-hexanol	1-hexanol	-	0.27	-
**15**	1037	1025	*p*-cymene	*1-methyl-4-propan-2-ylbenzene*	3.37	-	-
**16**	1062	1072	hotrienol	*3,7-Dimethyl-1,5,7-octatrien-3-ol*	2.51	-	-
**23**	1103	1083	nonanal	nonanal	0.97	0.15	-
**24**	1106	1108	2,2,6-trimethyl-3-keto-6-vinyltetrahydropyran	2,2,6-trimethyl-3-keto-6-vinyltetrahydropyran	-	0.61	-
**25**	1107	1167	2-nonen-1-ol	2-nonen-1-ol	0.65	0.72	-
**45**	1307	1331	silphiperfol-5-ene	*(1S,5R,8R,9S)-2,3,5,9-tetramethyltricyclo[6.3.0.0^{1,5}]undec-3-ene*	-	-	0.74
Monoterpene hydrocarbons							
**6**	897	902	santolina triene	*3-ethenyl-2,5-dimethylhexa-1,4-diene*	2.48	-	-
**8**	926	925	3-thujene	*2-methyl-5-propan-2-ylbicyclo[3.1.0]hex-2-ene*	0.36	-	-
**9**	937	933	α-pinene	*2,6,6-trimethylbicyclo[3.1.1]hept-2-ene*	1.11	-	-
**10**	955	946	camphene	*2,2-dimethyl-3-methylidenebicyclo[2.2.1]heptane*	1.95	-	-
**11**	981	973	β-pinene	*6,6-dimethyl-2-methylidenebicyclo[3.1.1]heptane*	0.82	-	-
**17**	1067	1060	γ-terpinene	*1-methyl-4-propan-2-ylcyclohexa-1,4-diene*	0.29	0.31	-
**18**	1075	1070	*cis*-sabinene hydrate	*2-methyl-5-propan-2-ylbicyclo[3.1.0]hexan-2-ol*	2.38	0.53	-
**20**	1079	1079	Terpinolene	*1-methyl-4-propan-2-ylidenecyclohexene*	0.18	-	-
**22**	1091	1074	*p*-cymenene	*1-methyl-4-propan-2-ylbenzene*	-	-	1.54
Oxygenated monoterpenes							
Alcohols							
**13**	1004	1000	2,5,5-trimethyl-3,6-heptadien-2-ol	2,5,5-trimethyl-3,6-heptadien-2-ol	0.43	-	0.47
**19**	1076	1072	artemisia alcohol	*3,3,6-trimethylhepta-1,5-dien-4-ol*	-	-	0.66
**21**	1086	1086	linalool	*3,7-dimethylocta-1,6-dien-3-ol*	1.53	-	-
**26**	1115	1207	carveol	*2-methyl-5-prop-1-en-2-ylcyclohex-2-en-1-ol*	2.05	-	-
**27**	1128	1105	fenchol	*1,3,3-trimethylbicyclo[2.2.1]heptan-2-ol*	0.46	0.27	-
**28**	1132	1126	*p*-menth-2-en-1-ol	*1-methyl-4-propan-2-ylcyclohex-2-en-1-ol*	1.79	0.33	-
**30**	1170	1181	myrtenol	*(6,6-dimethyl-2-bicyclo[3.1.1]hept-2-enyl)methanol*	0.97	-	-

32	1150	1180	isopinocarveol	*6,6-dimethyl-2-methylidenebicyclo[3.1.1]heptan-3-ol*	-	0.93	0.30
33	1182	1167	endo-borneol	*1,7,7-trimethylbicyclo[2.2.1]heptan-2-ol*	2.23	1.11	-
34	1189	1182	terpinen-4-ol	*4-methyl-1-propan-2-ylcyclohex-3-en-1-ol*	0.66	0.78	-
37	1233	1237	*cis*-geraniol	*(2E)-3,7-dimethylocta-2,6-dien-1-ol*	-	0.90	-
		Epoxides					
12	991	1017	myroxide	*2,2-Dimethyl-3-(3-methylpenta-2,4-dien-1-yl)oxirane*	1.57	-	-
		Ethers					
14	1037	1022	1,8-cineole	*1,3,3-trimethyl-2-oxabicyclo[2.2.2]octane*	1.35	0.13	1.49
41	1274	1250	geranyl vinyl ether	*(2E)-1-ethenoxy-3,7-dimethylocta-2,6-diene*	0.32	-	-
		Aldehydes and Ketons					
29	1156	1146	camphor	*1,7,7-trimethylbicyclo[2.2.1]heptan-2-one*	1.80	56.83	49.36
31	1146	1164	*α*-pinocarvone	*6,6-dimethyl-2-methylidenebicyclo[3.1.1]heptan-3-one*	-	0.22	-
35	1205	1171	myrtenal	*6,6-dimethylbicyclo[3.1.1]hept-2-ene-2-carbaldehyde*	1.92	-	-
40	1265	1261	6-isopropyl-3-methyl-7-oxabicyclo[4.1.0]-heptan-2-one	6-isopropyl-3-methyl-7-oxabicyclo[4.1.0]-heptan-2-one	1.05	-	1.20
42	1281	1272	*p*-mentha-1,8-dien-3-one]	*(6S)-3-methyl-6-prop-1-en-2-ylcyclohex-2-en-1-one*	0.12	-	-
		Esters					
38	1238	1305	myrtenyl acetate	(6,6-dimethyl-2-bicyclo[3.1.1]hept-2-enyl)methyl acetate	1.81	-	-
39	1246	1260	lyratyl acetate	*[(2E)-4-ethenyl-2,5-dimethylhexa-2,5-dienyl] acetate*	0.30	-	-
43	1288	1285	bornyl acetate	*(1,7,7-trimethyl-2-bicyclo[2.2.1]heptanyl) acetate*	1.28	-	-
46	1319	1350	*α*-terpinyl acetate	*2-(4-methylcyclohex-3-en-1-yl)propan-2-yl acetate*	0.27	-	-
		Other oxygenated derivatives					
36	1215	1192	(*Z*)-piperitol	*4-[(3S,3aR,6S,6aR)-3-(1,3-benzodioxol-5-yl)-1,3,3a,4,6,6a-hexahydrofuro[3,4-c]furan-6-yl]-2-methoxyphenol*	0.18	-	-
44	1289	1287	safrole	*5-prop-2-enyl-1,3-benzodioxole*	-	0.25	-
	Sesquiterpene hydrocarbons						
47	1353	1398	β-elemene	*(1S,2S,4R)- 1-ethenyl-1-methyl-2,4-bis(prop-1-en-2-yl)cyclohexane*	1.06	-	-
48	1363	1399	cyperene	*4,10,11,11-tetramethyltricyclo[5.3.1.0^{1,5}]undec-4-ene*	3.22	-	-
49	1378	1376	*α*-copaene	*1,3-dimethyl-8-propan-2-yltricyclo[4.4.0.0^{2,7}]dec-3-ene*	0.22	-	-
50	1392	1398	ciclohexane, 1-ethenyl-1-methyl-2,4-bis(1-methylethenyl)	ciclohexane, 1-ethenyl-1-methyl-2,4-bis(1-methylethenyl)	2.13	-	-

51	1426	1419	caryophyllene	*4,11,11-trimethyl-8-methylidenebicyclo[7.2.0]undec-4-ene*	1.99	-	-
52	1462	1496	*cis*-α-bisabolene	*1-methyl-4-[(2Z)-6-methylhepta-2,5-dien-2-yl]cyclohexene*	0.53	-	-
54	1482	1483	α-curcumene	*1-methyl-4-(6-methylhept-5-en-2-yl)benzene*	0.95	-	-
56	1502	1405	longifolene	*3,3,7-trimethyl-8-methylidenetricyclo[5.4.0.0^{2,9}]undecane*	0.55	-	-
57	1516	1433	γ-elemene	*(1S,2R,4R)-1-ethenyl-1-methyl-2,4-bis(prop-1-en-2-yl)cyclohexane*	0.61	-	0.79
58	1523	1524	δ-cadinene	*(1R,8aS)-4,7-dimethyl-1-propan-2-yl-1,2,3,5,6,8a-hexahydronaphthalene*	0.63	-	-
59	1539	1532	cubenene	(1*S*,4*R*,4*aS*)-1,6-dimethyl-4-propan-2-yl-1,2,3,4,4*a*,7-hexahydronaphthalene	0.11	-	-
61	1549	1542	α-calacorene	*(1S)-4,7-dimethyl-1-propan-2-yl-1,2-dihydronaphthalene*	4.92	0.27	-
	Oxygenated sesquiterpenes						
		Alcohols					
60	1546	1576	spathulenol	*(1aR,4aR,7S,7aR,7bR)-1,1,7-trimethyl-4-methylidene-1a,2,3,4a,5,6,7a,7b-octahydrocyclopropa[h]azulen-7-ol*	0.45	-	-
64	1579	1586	ledol	*(1aR,4R,4aS,7R,7aS,7bS)-1,1,4,7-tetramethyl-2,3,4a,5,6,7,7a,7b-octahydro-1aH-cyclopropa[e]azulen-4-ol*	2.05	-	-
67	1642	1637	caryophylladienol	*(5R)-10,10-dimethyl-2,6-dimethylidenebicyclo[7.2.0]undecan-5-ol*	5.92	2.19	-
68	1654	1638	isospathulenol	*1,1,4,7-tetramethyl-2,3,5,6,7a,7b-hexahydro-1aH-cyclopropa[h]azulen-7-ol*	0.86	-	-
70	1672	1681	α-santalol	*(Z)-5-[(1R,3R,6S)-2,3-dimethyl-3-tricyclo[2.2.1.0^{2,6}]heptanyl]-2-methylpent-2-en-1-ol*	0.79	0.98	0.50
71	1688	1694	β-santalol	*(Z)-2-methyl-5-[(1S,2R,4R)-2-methyl-3-methylidene-2-bicyclo[2.2.1]heptanyl]pent-2-en-1-ol*	1.36	-	-
74	1757	1713	farnesol	*3,7,11-trimethyldodeca-2,6,10-trien-1-ol*	5.92	28.83	-
75	1783	1778	costol	*2-(4a-methyl-8-methylidene-1,2,3,4,5,6,7,8a-octahydronaphthalen-2-yl)prop-2-en-1-ol*	1.15	0.58	0.80
76	1788	1701	shyobunol	*3-ethenyl-3-methyl-6-propan-2-yl-2-prop-1-en-2-ylcyclohexan-1-ol*	-	-	1.92
78	1795	1695	7-isopropyl-4,10-dimethylenecyclodec-5-enol	7-isopropyl-4,10-dimethylenecyclodec-5-enol	1.39	-	-
79	1822	1763	*cis*-lanceol	*(2E)-2-methyl-6-(4-methylcyclohex-3-en-1-yl)hepta-2,6-dien-1-ol*	0.36	0.43	-
80	1838	1777	15-hydroxy-α-muurolene	*2-[(1R)-4,7-dimethyl-1,2,4a,5,6,8a-hexahydronaphthalen-1-yl]propan-1-ol*	0.34	-	-
		Aldehydes and Ketons					
55	1496	1499	eremophilia-1(10),11-diene	*8,8a-dimethyl-1,3,4,6,7,8-hexahydronaphthalen-2-ylidene]propanal*	1.62	-	-

65	1595	1632	lLongiverbenone	*2,6,6,11-tetramethyltricyclo[5.4.0.$0^{2,8}$]undec-10-en-9-one*	2.00	0.44	-
72	1707	1693	germacra-3,7(11),9-trien-6-one	*(3E,7E)-3,7-dimethyl-10-propan-2-ylidenecyclodeca-3,7-dien-1-one*	0.44	-	-
77	1790	*1724*	thujopsenal	*4a,8,8-Trimethyl-1,1a,4,4a,5,6,7,8-octahydro-cyclopropa[d]naphthalene-2-carbaldehyde*	-	-	2.23
81	1853	1844	hexahydrofarnesyl acetone	*6,10,14-trimethylpentadecan-2-one*	1.89	-	-
82	1869	1867	α-santalone	(E)-6-(2,3-dimethyltricyclo[2.2.1.02,6]heptan-3-yl)-3-methylhex-3-en-2-one	-	-	21.58
		Esters					
53	1470	1510	*trans*-verbenyl isovalerate	*4,6,6-Trimethyl-bicyclo[3.1.1]hept-3-en-2-yl 3-methylbutanoate*	-	-	0.84
		Epoxides					
62	1551	1581	caryophyllene oxide	*4,12,12-trimethyl-9-methylidene-5-oxatricyclo[8.2.0.$0^{4,6}$]dodecane*	-	-	-
63	1570	1572	8-acetoxycarvo-tanacetone		2.08	-	-
66	1622	1606	humulene epoxide	*(4E,7E)-1,5,9,9-tetramethyl-12-oxabicyclo[9.1.0]dodeca-4,7-diene*	0.29	-	-
69	1665	1672	aromadendrene oxide (I)	1,1,7-trimethylspiro[2,3,4*a*,5,6,7,7*a*,7*b*-octahydro-1*aH*-cyclopropa[e]azulene-4,2'-oxirane	1.30	-	-
73	1716	1678	aromadendrene oxide (II)	*1,1,7-trimethylspiro[2,3,4a,5,6,7,7a,7b-octahydro-1aH-cyclopropa[e]azulene-4,2′-oxirane*	1.43	-	-
				**Oil Yields (%)**	**0.35**	**0.09**	**0.05**
				**Total Identified**	**89.67**	**100.00**	**100.00**
				**Non-terpenic derivatives**	**9.42**	**3.18**	**16.32**
				**Monoterpene hydrocarbons**	**10.02**	**1.32**	**2.01**
				**Oxygenated monoterpenes**	**21.64**	**61.51**	**53.01**
				**Sesquiterpene hydrocarbons**	**18.44**	**0.27**	**0.79**
				**Oxygenated sesquiterpenes**	**30.15**	**33.72**	**27.88**

The main common compounds are highlighted in grey colour.

LRI[a] = Linear Retention Index, experimentally obtained on a VF-5MS column using a C_7-C_{30} mixture of *n*-alkanes.

LRI[b] = Linear Retention Index as reported in NIST databases.
